# The Positive and Negative Suicidal Ideation Inventory among Portuguese Adolescents: Factor Structure and Gender Invariance

**DOI:** 10.3390/ejihpe14040065

**Published:** 2024-04-10

**Authors:** Marta Brás, Ana Cunha, João Antunes, Cláudia Carmo

**Affiliations:** 1 Faculty of Human and Social Sciences, University of Algarve, Campus de Gambelas, 8005-139 Faro, Portugal; ana.margarida.cunha.13@gmail.com (A.C.); jpantunes@ualg.pt (J.A.); cgcarmo@ualg.pt (C.C.); 2 Psychology Research Center, Rua de Santa Marta, 47-3°, 1169-023 Lisboa, Portugal

**Keywords:** adolescents, positive and negative suicidal ideation, confirmatory factor analysis, invariance

## Abstract

Suicide worldwide is an issue that needs to be addressed, and adolescents are an at-risk group. Assessing suicidal ideation is central to tackling the issue of suicide. The Positive and Negative Suicidal Ideation inventory is a widely validated measure of suicidal ideation, and yet, very little is known about its invariance across various groups. The present study aimed to adapt and test the PANSI’s structure in a Portuguese sample while testing its gender invariance. A total of 750 middle and high school students were recruited for the study, and data were collected on various suicide risk and protective factors, including the Portuguese-translated PANSI. Data were put through exploratory and confirmatory factor analysis. Kaiser’s criterion and scree plot both extracted two factors (64.10% variance explained). Confirmatory factor analysis also supported the PANSI’s structure (TLI = 0.943). The PANSI showed good reliability (α ≥ 0.83) and good construct and discriminative validity. The PANSI also exhibited scalar, but not strict, invariance. Overall, these results were similar to previous versions of this scale. The PANSI is a reliable measure of suicide risk among Portuguese adolescents. Future studies should further replicate these results in other cultures and expand on them by testing for invariance across other demographic variables.

## 1. Introduction

Suicide is the act of deliberately ending one’s own life [1], ranking among the top twenty global causes of death and accounting for approximately 700,000 deaths annually [2]. Suicide, particularly among young people, is a worldwide public health issue, standing as one of the three leading causes of death among youth [3]. Teens are a particularly vulnerable population due to developmental challenges faced during adolescence [4], as well as environmental factors often faced by this age group, such as bullying [3].

Evaluation and assessment are central in suicide prevention [5], and, as such, suicidal ideation data are often collected both in public health and population-based studies [6]. Suicide is often preceded by a series of thoughts and behaviors. Pre-suicidal thoughts can span an entire range from an occasional thought about death to considering and planning a suicide attempt. These thoughts and beliefs are often categorized as suicidal ideation [1] and are particularly common among adolescents [7]. Suicidal ideation stands out as a precursor to self-harming and/or suicidal behaviors, serving as the initial step in the suicidal process [8].

In Portugal, suicide is a public health problem, and adolescents are considered an at-risk group [9], particularly in the Autonomous Region of the Azores (ARA), where it has been shown that a relevant amount of young people exhibit suicidal ideation and/or self-harm [10,11], as well as associated risk factors [12]. Therefore, data show that the Portuguese suicidal context is notable, with the context of the ARA being particularly alarming regarding youth [13].

Worldwide, various tools have been developed to measure ideation-based suicide risk factors among youth, such as the Beck Depression Inventory [14] or the Center for Epidemiological Studies Depression Scale for Children [15]. Although these are useful tools, these, and most other suicide assessment tools, share the limitation that they focus on suicide risk and ideation through measures of thoughts and actions related to an intention to die, neglecting protective factors. This is important in assessment because evidence suggests various interaction effects between risk and protective factors and demographic factors [12,16], and it is also important in clinical practice, as interventions must promote protective factors and reduce risk factors [17]. Although there are some measures of protective factors, such as the Reasons for Living Inventory [18,19,20], very few measures consider risk and protective factors simultaneously [21].

The Positive And Negative Suicidal Ideation (PANSI; Ref. [21]) inventory is a widely validated measure of positive and negative suicidal ideation, meaning that it accesses ideation related to suicide risk, such as hopelessness and a lack of meaning in life, aggregated into negative ideation, and protective ideation, such as the perceived ability to cope with life’s problems or control over one’s own future, aggregated into positive ideation. In Portugal, the psychometric characteristics of the PANSI have been studied in young adults [22] but not in adolescents. The PANSI has been adapted for various cultures (e.g., Villalobos-Galvis [23]) and populations (e.g., Osman et al. [24]), consistently retaining its two-factor structure spread across fourteen items, including in children and adolescents [25,26,27,28,29]. The PANSI shows particular utility due to its widespread validation, its multidimensional assessment, and its briefness (14 items), leading to ease of application. Previous studies have investigated the construct validity of the PANSI through its relationship with depressive symptoms (e.g., [21,28]) and hopelessness (measured by the Beck Hopelessness Scale [21,23,28,29,30,31])—showing a positive correlation with negative suicidal ideation—and with reasons for living (measured by the Reasons for Living Inventory [24,28,30,31])—showing a positive correlation with positive suicidal ideation.

As it stands, using the PANSI in the assessment of suicide risk is particularly advantageous due to its ability to give a multifaceted assessment by also capturing protective factors, instead of the common approach that focuses solely on assessing risk factors [27]. Another advantage the PANSI might present is related to its invariance across gender.

The suicidal process is not experienced equally between boys and girls, as evidenced by the presence of different suicide risks across gender [32]. Recognizing and addressing group differences is essential to promoting equity in public health and research, and failing to do so can negatively affect boys and, especially, girls [33], as is evidenced by a lack of data regarding the risk of death by suicide for girls [32]. Chen et al. [27] showed strict invariance across gender in a Chinese adolescent sample, demonstrating the PANSI’s lack of gender bias in its factor structure. Reproducing and expanding on these results is the next step in promoting an equitable practice in this field and, as such, the present study aims to achieve the following objectives: (1) to assess the reliability and validity of the PANSI scale in a non-clinical sample of Portuguese adolescent students; (2) assess the internal structure of the PANSI through confirmatory factorial analysis; and (3) test the measurement invariance of the PANSI scale across gender.

## 2. Materials and Methods

### 2.1. Participants

A total of 750 Portuguese individuals from middle and high schools in the autonomous region of the Azores participated in the present study by filling an evaluation battery in the classroom. After outliers were removed (*p*_Mahalanobis d-squared_ ≤ 0.001; 64 cases removed), based on PANSI scores, the final sample comprised 686 students (M_age_ = 14.68; *SD* = 1.84), and was 51% female; 74.9% were in middle school and 25.1% were in high school. A total of 57 participants (8.31%) had made at least one suicide attempt in the past, and further information can be viewed in the Results section.

This final sample was randomly assigned to either exploratory or confirmatory factor analysis using the Select Cases function in SPSS to randomly extract approximately 50% of cases from the full sample for each condition. Exploratory factor analysis (EFA) had 345 cases and confirmatory factor analysis (CFA) had 341 cases, and groups did not differ by gender (*p* = 0.171) or school level (*p* = 0.064) but differed by age (*p* = 0.019), although this was a small difference (Cohen’s *d* = 0.180).

### 2.2. Measures

A sociodemographic questionnaire was handed out. This questionnaire asked participants to report their age, current grade, gender, and a yes/no question as to whether the participant themself had made at least one suicide attempt in the past.

The Positive and Negative Suicidal Ideation Inventory (PANSI; Osman et al. [21]; originally adapted to adolescents by Osman et al. [28]) comprises 14 items assessed using a 5-point Likert-type scale (1 = Completely Disagree; 5 = Completely Agree). Within its structure, 8 items gauge negative suicidal ideation, while 6 items capture positive ideation, and both aspects demonstrate robust internal consistency, with α values of 0.94 and 0.81, respectively.

The Beck Hopelessness Scale (BHS; Beck et al. [34]; adapted to the Portuguese population by Cruz [35]) contains 20 True/False hopelessness-related statements intended to measure suicide risk through hopelessness. The scale showed good reliability in the original (α = 0.93) and Portuguese (α = 0.93) versions. In the present study, the BHS showed acceptable reliability (α = 0.78).

The Center for Epidemiological Studies Depression Scale for Children (CES-DC; Weissman [15]; adapted by Carvalho et al. [36]) is a 20-item measure of the frequency of depressive symptoms, rated from 0–3. The overall scale showed good reliability (α = 0.90) in the Portuguese version and the present sample (α = 0.92).

The Reasons for Living Inventory for Adolescents (RFL-A; Osman et al. [18]; adapted by Brás et al. [19]) measures reasons for living by 32 items rated on a Likert-type scale (1 = Not Important; 6 = Extremely Important). It shows excellent reliability in both its original (α = 0.96) and Portuguese (α = 0.94) versions, which was replicated in this study sample (α = 0.97).

### 2.3. Translation

After obtaining consent from the original authors, the translation procedure employed a backward–forward method of translation to ensure the content’s conceptual and cross-cultural equivalence [37]. A native-speaking professor proficient in English conducted the initial translation into Portuguese. Subsequently, a psychologist, fluent in both languages and unfamiliar with the original PANSI, performed the backward translation into English. Ultimately, a panel consisting of four independent bilingual experts, well-versed in the instrument, scrutinized all three scales. They rectified any disparities between the original version and the back-translated English version, refining the Portuguese rendition accordingly.

### 2.4. Data Collection

Data were gathered by directly contacting the various schools on the island of São Miguel in the autonomous region of Azores, Portugal, leading to seven schools accepting to collaborate with the present study. The school staff were then entrusted with informing the student population of the study and handing out consent forms to the students, giving them at least two weeks to give their parents the consent form and then bring it back to school. At a later date, a researcher was present at the school to apply the questionnaire to the students whose parents had consented to the study and clarify any questions that they may have. The questionnaire was only applied to students of Portuguese nationality who were able to read well enough to understand the questions (in this last selection criteria, teachers were asked to inform the responsible researcher if they believed the student might struggle with understanding the questionnaire, but there were no such cases). Participants were also to be removed from the study if there was evidence of non-compliance (giving the same score in all answers) or if there were too many missing values (>20%) in any one questionnaire, but, again, there were no such cases.

### 2.5. Data Analysis

Descriptive and correlational analyses of the data were performed with the full sample using SPSS v. 29.00.00. Missing data were below 20% and were, therefore, corrected through imputation (Expectation Maximization method). Reliability was measured with Cronbach’s α. Exploratory factor analysis was also performed with SPSS v. 29.00.00. The number of factors was selected based on analysis of Eigenvalues, scree plots, and the loadings of the rotated solution. The selected extraction method was Principal Components, and an Orthogonal Rotation (Varimax) was selected.

AMOS version 29 was utilized for confirmatory factor analysis (CFA). Estimation employed the maximum likelihood method, supplemented by bootstrapping (Bollen-Stine, 500 samples) to address non-normality (multivariate kurtosis = 201.60; critical ratio = 87.94). Fit index assessments followed Kline [38] and Byrne [39]. As such, the χ^2^/df ratio should range between 1 and 2, with the Tucker–Lewis Index (TLI) and the Comparative Fit Index (CFI) deemed acceptable at 0.90 and excellent at 0.95 or higher. The Root-Mean-Square Error of Approximation (RMSEA) and the Standardized Root-Mean-Square Residual (SRMSR) are considered favorable below 0.05 and 0.08, respectively. Finally, invariance testing followed Cheung and Rensvold’s [40] standard of ∆TLI and ∆CFI ≤ 0.01 and ∆RMSEA ≤ 0.015, which were indicative of invariance between models.

Convergent validity was assessed using average variance extracted (AVE), which, ideally, should be above 0.50, according to Fornell and Larcker’s [41] criterion, and multicollinearity was assessed by checking if inter-item correlations (*r*) were <0.85 [38]. Discriminative validity was assessed by testing each subscale’s ability to accurately predict one previous suicide attempt among the participants using binary logistic regression.

## 3. Results

### 3.1. Descriptive Statistics

Descriptive statistics for the main variables of the study, divided according to demographic information, can be found in Table 1. The results are divided by learning level, with high school (grades 10 through 12) being split between regular curriculum and career-focused curriculums.

Among the present variables, statistically significant (*p* ≤ 0.001) gender differences were found for PANSI scores (both subscales), CES-DC scores, and suicide attempts.

### 3.2. Exploratory Factor Analysis

Before beginning EFA, preliminary analyses showed that there were no issues with the sample related to sphericity (Bartlett’s test: chi-square = 3060.79; *df* = 91; *p* ≤ 0.001), sampling adequacy (KMO = 0.904), or collinearity (*r* < 0.80).

Based on Kaiser’s criterion (rotated Eigenvalue for two components= 3.26; variance explained = 64.10%) and on the analysis of the scree plot, two factors were retained. All items loaded onto one of the two factors, and none loaded significantly with both factors, although Item 13 had a double loading in the unrotated solution, using the criteria of loadings superior to 0.45 in both cases, in accordance with the original adaptation of the PANSI. As expected, all positive items were loaded onto PANSI-PI, and all negative items were loaded onto PANSI-NSI. Table 2 displays further information on EFA results.

### 3.3. Confirmatory Factor Analysis

In previous research, Sinniah et al. [31] elected to correlate the errors of Items 3 and 4, as well as those of Items 10 and 11, within their model, based on empirical and theoretical considerations. The same procedure was employed in the present study. Both the CFI (0.961) and TLI (0.953) were within the good to excellent range. The RMSEA (0.079; 90%CI: 0.067; 0.090) did not fall within ideal standards, but scores of ≤0.80 are considered acceptable, and SRMR (0.077) was within the desired values. χ^2^/*df* (230.07/74= 3.11) was outside the acceptable range, although it is important to point out that this measure is highly sensitive to sample size [38].

### 3.4. Validity and Reliability Analysis

Standardized factor loadings and internal consistency metrics are presented in Table 3. AVE in negative ideation was 0.78 and for positive ideation, it was 0.48. Inter-item correlations for positive ideation varied between 0.26 and 0.63, while in negative ideation, the range was 0.52–0.86, which is above Kline’s [38] threshold of <0.85, although there was only one correlation that surpassed this limit. Reliability was excellent for negative ideation (α = 0.95) and good for positive ideation (α = 0.83).

### 3.5. Construct Validity

All correlations were statistically significant and went in the expected direction. PANSI-NSI correlated positively with BHS (*r* = 0.42; *p* ≤ 0.001) and CES-DC (*r* = 0.61; *p* ≤ 0.001) and negatively with RFL-A (*r* = −0.36; *p* ≤ 0.001) and PANSI-PI (*r* = −0.24; *p* ≤ 0.001). Inversely, PANSI-PI was negatively correlated with BHS (*r* = −0.45; *p* ≤ 0.001) and CES-DC (*r* = −0.37; *p* ≤ 0.001) and positively with RFL-A (*r* = 0.39; *p* ≤ 0.001).

### 3.6. Discriminative Validity

The overall classification accuracy of the negative ideation subscale was 93.3%, and the Omnibus Coefficient Model test (χ^2^ = 111.70; *df* = 1; *p* ≤ 0.001) supported the model, although the Hosmer/Lemeshow Test (χ^2^ = 12.26; *df* = 2; *p* = 0.002) was less conclusive (ideally, *p* > 0.05). The overall classification accuracy of the positive ideation subscale was 91.7%, and both tests supported the model (Omnibus Coefficient Model: χ^2^ = 13.64; *df* = 1; *p* ≤ 0.001; Hosmer/Lemeshow Test: χ^2^ = 8.32; *df* = 7; *p* = 0.305). Table 4 represents the main indicators for each logistic regression in positive and negative ideation. Each subscale, independently, significantly predicted a previous suicide attempt in the expected direction.

### 3.7. Gender Invariance

Model fit indexes pertaining to invariance measurements can be found in Table 5. Boys were set as the reference group, and Model 1 shows fit indexes for the base model, with no constrictions applied. To test metric invariance, factor loadings were set to be equal between groups (Model 2). ∆TLI and ∆CFI both remained ≤0.01 and ∆RMSEA ≤ 0.015 when compared to the previous model, indicating that metric invariance was confirmed. To test scalar invariance, Model 3 had the added restriction of assuming that item intercepts are invariant across groups; therefore, ∆TLI, ∆CFI, and ∆RMSEA are below the threshold, indicating scalar/strong invariance. In Model 4, residual variances were set to be equal between groups, ∆TLI and ∆CFI > 0.01, although ∆RMSEA ≤ 0.015, indicating that the PANSI factor structure was not stable enough under this restriction to ascertain strict invariance for gender. This version of the PANSI shows strong, but not strict, gender invariance.

## 4. Discussion

As was mentioned previously, boys and girls tend to experience pre-suicidal symptoms differently [42], which was consistent with the finding that average scores in CES-DC and PANSI were different between boys and girls, as was the number of suicide attempts. Considering these differences, it was important to verify if current suicidal ideation can be interpreted equally among genders. The present study aimed to replicate previous research regarding gender invariance in the children’s version of PANSI, while also validating its Portuguese version. EFA results supported a two-factor structure of the PANSI, similar to previous versions of the scale [28]. During CFA, all fit indexes fell within acceptable values or better, with the exception of χ^2^/df. Although, ideally, all values would fall within the desired ranges; this last criterion is considerably less robust than all the other ones considered [38], indicating that the Portuguese version of the PANSI shows an appropriate factor structure.

The scale also showed good validity and reliability overall. Although reliability scores raise some concerns regarding Item 4 slightly raising the scale’s α, the literature regarding the adolescent version of the PANSI shows that its items do not factor as well into their respective scales, as is the case with Avendaño-Prieto et al. [25], who found that certain items tend to aggregate into a third factor. Additionally, regarding positive ideation’s lower reliability, Chen et al. [27] argue that non-clinical samples might have a harder time understanding some of the items relating to this subscale. These results, in addition to positive ideation’s relatively low AVE, highlight the difficulty of assessing suicidal protective factors through self-report in non-clinical populations. Future researchers who choose to employ this version of the PANSI in clinical populations should be careful when interpreting results, as further validation in clinical adolescent samples is needed.

Discriminative validity results show that the scale has robust discriminative validity, replicating previous results (e.g., Avendanõ-Prieto et al. [25]). The PANSI’s construct validity was also confirmed and replicated previous results, for example, correlational results with the BHS showed significant and stronger correlations with both PANSI scales when compared to other adaptations [24,29], and the RFL-A showed similar correlations to Osman et al. [24]. Comparative results show that assessments of negative suicidal ideation are comparable to hopelessness and depression, as expected, since all these constructs are identifiable suicide risk factors [15,30,33]. The positive ideation subscale’s positive correlation with reasons for living was also expected. More importantly, the differentiated correlations between these subscales and the measures used for validity testing show that positive suicidal ideation is more than a lack of negative suicidal ideation, it is a distinguishable construct [28].

Invariance results indicate that the PANSI has strong gender invariance in this population. Although these results are not as conclusive as the strict invariance found by Chen et al. [27], these results are sufficient to make meaningful comparisons between groups, and it can be considered that they share the same operational definition [40]. There are various possible explanations for the differences between these and Chen et al.’s [27] results. Firstly, this disparity may point to an interaction effect between gender and culture [43], which might imply that other versions of the PANSI might show stronger or weaker invariance, depending on culture. If such a hypothesis is confirmed, these findings could have implications about how children are socialized and how they impact childrens’ and adolescents’ “relationship” with death. Alternatively, the fact that both this and Chen et al.’s [27] sample comprises students could imply that educators might also play a role in this process, as they have been shown to influence students’ suicidal attitudes [44]. It would be interesting to investigate if these teachers take a differentiated approach for boys and girls in this regard and if this is a cause or an outcome of the current gender differences found for suicidal ideation. Future studies should continue to test the scale’s invariance across cultures.

## 5. Conclusions

The PANSI scale shows solid psychometric properties and is, therefore, an appropriate measure of suicide risk among Portuguese youth. Additionally, this research adds to the evidence that supports this brief scale as a suitable tool for negative suicidal ideation, while raising some concerns regarding the suitability of the use of its positive suicidal ideation subscale for non-clinical populations. Although still acceptable, positive suicidal ideation showed weaker psychometric properties, indicating that the construct of positive suicidal ideation might be difficult to understand for non-clinical adolescents. Regardless, both positive and negative ideation accurately predicted a previous suicide attempt, indicating that the PANSI shows clinical utility in the present population.

These results are particularly relevant as the accurate measurement of suicidal ideation in the studied population may be a valuable contribution to both prevention and remediation efforts in interventions with this population. Considering the impact suicidal ideation has in the context of Portuguese youth suicidality [10,12,45], it should be taken as a central variable in the conception of practical actions within this group, such as the development of clinical intervention programs and or/protocols, community interventions, health policies, or other means of intervention.

Invariance results support a similar structure of the PANSI between genders. The implication of such a finding is that the PANSI can be used to assess and imply meaningful differences or similarities between genders in relation to thoughts about death and one’s desire to survive. Caution is advised when generalizing these data, as the sample of adolescents is from a particular cultural context, although the sample size is large. Further studies should be conducted to explore the differences found between the present invariance results and the previous literature. Discovering if the PANSI’s gender invariance is context dependent could be valuable in the assessment of suicidality and could have implications for clinicians and educators.

This study is limited by its inability to access certain variables that would be valuable both in validating this scale from the standpoint of equity and psychometrics and by the reduced number of previous studies that could serve as comparison points in the interpretation of the present results. One other limitation concerns the sample’s representativeness due to the fact that only participants whose parents consented to the study could participate could introduce bias into the results, although this issue is ubiquitous to most studies that rely on self-report. Future studies could address these concerns by testing this scale’s test–retest reliability and by testing invariance on other demographic variables, such as ethnic background, for example.

## Figures and Tables

**Table 1:** Descriptive statistics for the study population.

Group	N		Age		PANSI Negative	PANSI Positive	BHS	RFL-A	CES-DC
	Total	Suicide Attempt	M (SD)	Min/Max	M (SD)	M (SD)	M (SD)	M (SD)	M (SD)
Full Population	686	57	14.68 (1.84)	12/19	1.24 (0.56)	3.30 (0.94)	3.98 (3.29)	4.83 (1.11)	16.75 (12.15)
EFA	345	38	14.85 (1.88)	12/19	1.25 (0.54)	3.27 (0.94)	4.07 (3.28)	4.79 (1.12)	17.09 (11.86)
CFA	341	19	14.52 (1.79)	12/19	1.24 (0.58)	3.33 (0.95)	3.89 (3.31)	4.86 (1.11)	16.41 (12.44)
Gender									
Boys	336	14	14.80 (1.81)	12/19	1.14 (0.40)	3.44 (0.95)	3.78 (3.21)	4.86 (1.13)	12.83 (9.49)
Girls	350	43	14.57 (1.87)	12/19	1.34 (0.67)	3.17 (0.92)	4.17 (3.36)	4.79 (1.09)	20.52 (13.19)
School Level									
Middle School	514	38	13.98 (1.43)	12/19	1.23 (0.54)	3.26 (0.97)	4.01 (3.16)	4.80 (1.13)	16.34 (11.92)
High School (Regular)	80	4	16.13 (1.10)	14/19	1.16 (0.43)	3.53 (0.77)	3.44 (2.92)	5.03 (0.82)	15.64 (11.33)
High School (Career)	92	15	17.34 (1.13)	15/19	1.40 (0.74)	3.31 (0.91)	4.27 (4.17)	4.76 (1.20)	20.01 (13.62)

**Table 2:** Rotated and unrotated factor loadings.

Item	Extracted Communality	Unrotated Factor Loadings		Rotated Factor Loadings	
		Factor 1	Factor 2	Factor 1	Factor 2
1	0.724	0.826	0.207	0.850	−0.038
2	0.420	−0.150	0.630	0.037	0.647
3	0.745	0.843	0.183	0.861	−0.065
4	0.449	0.662	0.109	0.665	−0.085
5	0.655	0.797	0.142	0.804	−0.091
6	0.614	−0.204	0.756	0.021	0.783
7	0.692	0.795	0.243	0.832	0.006
8	0.638	−0.203	0.773	0.026	0.798
9	0.800	0.887	0.118	0.884	−0.140
10	0.791	0.873	0.170	0.885	−0.086
11	0.782	0.865	0.186	0.882	−0.069
12	0.580	−0.302	0.699	−0.090	0.756
13	0.522	−0.467	0.551	−0.290	0.662
14	0.560	−0.376	0.647	−0.176	0.727

**Table 3:** Standardized factor loadings, total item correlations, and subscale alphas.

Item	Standardized Factor Loadings		Total Item Correlation		Alpha if Item Removed	
	Negative	Positive	Negative	Positive	Negative	Positive
2	--	0.49	--	0.48	--	0.83
6	--	0.70	--	0.65	--	0.80
8	--	0.70	--	0.65	--	0.80
12	--	0.75	--	0.66	--	0.79
13	--	0.73	--	0.58	--	0.81
14	--	0.73	--	0.61	--	0.80
1	0.91	--	0.84	--	0.94	--
3	0.91	--	0.85	--	0.94	--
4	0.73	--	0.66	--	0.96	--
5	0.85	--	0.79	--	0.95	--
7	0.92	--	0.84	--	0.94	--
9	0.92	--	0.88	--	0.94	--
10	0.90	--	0.87	--	0.94	--
11	0.91	--	0.87	--	0.94	--

**Table 4:** Logistic regression models for both PANSI subscales.

Model	Predictor	Β	S.E.	Sig.	OR (95% CI)
1	PANSI Negative	2.03	0.23	≤0.001	7.60 (4.86; 11.88)
	Constant	−5.42	0.40	≤0.001	0.004
2	PANSI Positive	−0.52	0.14	≤0.001	0.59 (0.45; 0.78)
	Constant	−0.78	0.43	0.070	0.458

**Table 5:** Fit statistic changes across models.

Model	χ^2^	*df*	TLI	CFI	SRMR	RMSEA	∆TLI	∆CFI	∆RMSEA
1	391.174	148	0.918	0.933	0.089	0.070	--	--	--
2	440.946	160	0.912	0.923	0.087	0.072	−0.006	−0.010	+0.002
3	465.933	172	0.914	0.919	0.087	0.071	+0.002	−0.004	−0.001
4	613.799	186	0.885	0.882	0.106	0.082	−0.029	−0.037	+0.011

## Data Availability

The data can be made available for consultation upon request from the corresponding author.
